# Orexins, Sleep, and Blood Pressure

**DOI:** 10.1007/s11906-018-0879-6

**Published:** 2018-07-10

**Authors:** Mariusz Sieminski, Jacek Szypenbejl, Eemil Partinen

**Affiliations:** 1 Department of Emergency Medicine, Medical University of Gdansk, Smoluchowskiego 17, 80-235 Gdansk, Poland; 2 Department of Neurology, University of Helsinki, Helsinki, Finland; 3 Vitalmed Helsinki Sleep Clinic, Helsinki, Finland

**Keywords:** Orexin, Hypocretin, Blood pressure, Sleep, Narcolepsy, Autonomic nervous system

## Abstract

**Purpose of Review:**

The aim of this review was to summarize collected data on the role of orexin and orexin neurons in the control of sleep and blood pressure.

**Recent Findings:**

Although orexins (hypocretins) have been known for only 20 years, an impressive amount of data is now available regarding their physiological role. Hypothalamic orexin neurons are responsible for the control of food intake and energy expenditure, motivation, circadian rhythm of sleep and wake, memory, cognitive functions, and the cardiovascular system. Multiple studies show that orexinergic stimulation results in increased blood pressure and heart rate and that this effect may be efficiently attenuated by orexinergic antagonism. Increased activity of orexinergic neurons is also observed in animal models of hypertension.

**Summary:**

Pharmacological intervention in the orexinergic system is now one of the therapeutic possibilities in insomnia. Although the role of orexin in the control of blood pressure is well described, we are still lacking clinical evidence that this is a possibility for a new approach in the treatment of cardiovascular diseases.

## Introduction

We are celebrating the twentieth anniversary of the discovery of hypocretins/orexins. Those two decades appeared to be extremely fruitful: We identified a new neuronal system within the central nervous system with projections to multiple areas of the brain and discovered its involvement in almost all crucial life processes. It was almost immediately discovered that orexins are responsible for the regulation of appetite; next, it became clear that they steer also the circadian rhythm of the sleep-wake cycle. Later, new facts emerged, showing their role in energy expenditure, locomotor activity, motivation and addiction, cognition, emotional control, and, last but not least, control of the cardiovascular system. With such a variety of functions, orexins appear to be a promising target for therapeutic interventions aimed at solving the most pivotal health problems of our civilization: psychiatric disorders, cognitive dysfunctions, addictions, insomnia, and diseases of the cardiovascular system. So far, we were able to introduce new treatment options for insomnia based on drugs that block the orexin system. This review presents the most recent, significant discoveries related to the role of orexins in the control of sleep and blood pressure.

## Orexins Uncovered: the Peptides, the Receptors, the Anatomy

It was in 1998, when simultaneously, independently, and with different purposes, the work of two groups of scientists led to the discovery of two novel neurotransmitters produced in the lateral part of the hypothalamus. De Lecea et al. decided to name the peptides hypocretin-1 and hypocretin-2 to indicate the *hypo*thalamus as the locus of their origin and to signify that they belong to the family of in*cretins* [1]. Sakurai and colleagues while seeking for a natural ligand for G-protein-coupled receptors described earlier also discovered two peptides produced in the lateral hypothalamus. As it was quickly observed that those peptides injected directly into the brains of rats led to an increase in the animals’ appetite, they were called orexin-A and orexin-B (from the Greek word *orexis*, meaning appetite) [2].

Orexin-A is composed of 33 amino acids, while orexin-B is a peptide that contains 28 amino acids. Both peptides are products of a proteolytic process using the common precursor peptide prepro-orexin. A gene localized on the chromosome 17 encodes this precursor peptide [3]. Expression of the *prepro-orexin* gene is dependent on numerous physiological factors like fasting status or sleep duration [4, 5].

Orexins interact with two G-protein-coupled receptors (GPCRs): OX1R and OX2R. Orexin-A has a similar affinity to both receptors while orexin-B preferentially binds to OX2R receptors. Binding of orexin to any of those receptors evokes an excitatory action, most frequently leading to a depolarization of the postsynaptic neuron. The electrical effect of the orexin receptor activation is a consequence of the inhibition of potassium channels, the opening of non-selective cation channels, and the influx of calcium ions [6, 7, 8••].

Orexins are exclusively produced by a population of no more than 80,000 hypothalamic neurons located in the lateral and dorsomedial parts of the hypothalamus as well as in the perifornical area [9]. Apart from orexins, those neurons also secrete dynorphin [10], glutamate [11], and neuronal activity-regulated pentraxin (NARP) [12]. Orexin neurons receive numerous efferents from cortical and limbic areas (e.g., prefrontal cortex, bed nucleus of stria terminals, or amygdala) as well as from brainstem regions (e.g., dorsal raphe nucleus, rostral ventrolateral medulla) [13–15]. The afferent projections of orexinergic neurons reach the ventral tegmental area, the nucleus accumbens, the basal forebrain, the locus coeruleus, the dorsal raphe nuclei, the amygdala, and the reticular formation [16•, 17, 18]. As it may be concluded from this diversity of brain areas interconnected with orexinergic neurons, they must play a role in many various processes.

## Mighty Transmitter—the Roles of Orexins

Immediately after its discovery, it became apparent that centrally injected orexin provokes food-seeking behavior in laboratory animals [2]. This observation was confirmed in further studies. Van Holst et al. demonstrated recently that orexin-deficient humans (patients with narcolepsy type I) more frequently reach for salty or sweet snacks compared to controls and to patients with idiopathic hypersomnia. It was especially interesting that these patients consumed snacks even after satiety and after being educated about which snacks should be avoided. These findings suggest that orexin regulates the caloric intake and disturbances in the action of this neuropeptide may be related to obesity, which is frequently observed in patients with narcolepsy [19•]. On the other hand, additional orexinergic stimulation, e.g., as a result from the injection of orexin into the ventral tegmental area, leads to increased food intake in laboratory animals. This observation is further supported by the finding of the same group that the specific OX1R antagonist SB334867 blocks the food intake [20]. González et al. observed that activity of orexinergic neurons is suppressed by the act of eating, i.e., the activity of these neurons is reduced during feeding regardless of the kind of food consumed by the laboratory animals, and complete lack of orexin signaling, e.g., in orexin knockout mice, leads to massive overeating and obesity [21]. These findings suggest that orexins play a dual role in the process of food intake: they induce eating behavior and simultaneously regulate the amount of food intake. This role of orexins in the metabolic balance of organisms is additionally reinforced by their influence on the level of physical activity. Kosse et al. recently discovered that activation of orexinergic neurons stimulates a population of GAD65-positive nerve cells (neurons containing glutamic acid decarboxylase 65) in hypothalamus, which in turn are responsible for increasing the mobility of laboratory animals [22]. This may suggest that orexins stimulate energy expenditure through increased mobility. Coborn and colleagues who performed microinjections of dual orexin receptor antagonist into the ventrolateral preoptic area of rats and observed a significant decrease in orexin-induced spontaneous physical activity as well as lower energy expenditure during sleep recently confirmed this hypothesis. In the same series of experiments, injected orexin-A led to a shortened sleep and an increased energy expenditure [23]. It seems that the metabolic role of orexin may be simply described by the sentence “eat as much as possible and burn it as quickly as you can”.

The most significant clinical role of orexin observed so far is the regulation of the diurnal sleep-wake rhythm. Deficiency of orexins leads to narcolepsy type 1, a disease manifesting in increased daytime somnolence, involuntary naps, disorganized sleep architecture, increased presence and shortened latency of REM sleep, and cataplexy, a sudden loss of muscle tone. In humans, narcolepsy is caused by an extremely reduced number of hypothalamic orexin neurons and deficiency of circulating orexin. These observations imply that orexins have stimulating, wake-promoting actions. This was recently confirmed by findings of Vassalli and Franken who showed electrophysiological evidence that orexins are necessary to sustain active, spontaneous wakefulness (during which an animal undertakes explorative or goal-oriented behaviors) [24].

The role of orexin in narcolepsy is well described and recently published papers show that they are also important factors in insomnia. Tang et al. found that orexin-A plasma levels were higher in patients with insomnia and correlated with their severity of insomnia [25]. The role of orexin in insomnia was clinically confirmed by the efficacy of orexin receptor antagonists in the treatment of insomnia. The first drug of this group shown in a series of clinical trials to be effective and safe in the therapy of insomnia was suvorexant and it is now approved for clinical use. Suvorexant is a dual orexin receptor antagonist. Its use promotes sleep in healthy subjects [26] and improves sleep efficiency in patients with insomnia [27]. In terms of subjective sleep quality assessment and polysomnographic parameters, it was effective in both a 3-month [28] and a 12-month [29] trial period. The drug proved to be safe and well-tolerated, with the most common adverse event being daytime sleepiness (reported by 13% of patients from the suvorexant group, compared to 3% in the placebo group) [29]. The therapy with the orexin receptor antagonist resulted in faster sleep onset and improved sleep maintenance, moreover with no acute symptoms of rebound or withdrawal after abrupt cessation of the treatment [30]. Vermeeren and colleagues performed a study in healthy volunteers aiming at an assessment of the impact of suvorexant on their ability to drive and found no clinically meaningful residual effect of suvorexant on next-morning driving [31]. Born et al. described a series of experiments performed in preclinical, animal models showing a probably low abuse potential of suvorexant [32]. Schoedel et al. compared the abuse potential of suvorexant and a GABA receptor modulator, zolpidem, in a population of recreational polydrug users, finding that although both drugs have similar abuse potential, the number of abuse-related adverse events was lower in the suvorexant group [33].

The efficacy and safety of suvorexant facilitated studies on new orexin receptor antagonists. Roth et al. described an increase in total sleep time, a reduction of sleep latency, and a decrease in the wake time after sleep onset in elderly patients taking almorexant—another dual orexin receptor antagonist. The drug was well-tolerated with an incidence of adverse events comparable to placebo [34]. Black et al. found similar results in a 2-week blind placebo- and zolpidem-controlled trial. Compared to classic hypnotics, the therapy with almorexant was not related to rebound or withdrawal phenomena. The improvements in objective sleep parameters were greater for almorexant than for zolpidem, although the trial was not conceptualized as a head-to-head study [35]. Filorexant is another dual orexin receptor antagonist shown to be effective and safe in a controlled clinical trial [36]. We shall expect further clinical trials with dual orexin receptor antagonists in insomnia, as new molecules are being discovered and characterized. Recently, Beuckmann et al. described pharmacologic features of lemborexant—their results suggest that the new molecule will have a therapeutic effect similar to suvorexant and almorexant [37]. As dual orexin receptor antagonists already have their place in the therapy of insomnia, the clinical community anticipates the introduction of selective orexin receptor antagonists. De Boer et al. recently published results of a study in which the efficacy of the OX2R antagonist seltorexant in the treatment of primary insomnia was compared to placebo. This placebo-controlled, crossover study showed that seltorexant significantly reduces sleep latency, improves sleep efficiency, increases total sleep time, and shortens wake time after sleep onset [38].

The sentence coined at the end of the first paragraph of this section should, therefore, be extended into the following one: “Stay awake to eat as much as possible and to burn it as quickly as you can”. To make the last part of this sentence real, another feature is necessary: motivation. Hypothalamic orexin neurons send multiple projections to mesolimbic structures involved in motivation and reward-seeking behaviors, such as the ventral tegmental area and the nucleus accumbens [39••]. Orexinergic stimulation promotes reward-seeking behaviors as it was shown in animal models of food rewards [40]. Consequently, orexin system is involved in drug abuse and addiction. It seems that postsynaptic actions of orexins evoke drug-seeking behaviors. This was demonstrated by studies using antagonists of orexin receptors. Martin-Fardon et al. showed recently that orexin neurons are active in cocaine-induced reward (drug)-seeking behavior [41]. Schmeichel et al. were able to reduce the motivation of rats for seeking both cocaine and palatable food by blocking the production of orexin [42]. Those results suggest that orexin is required for the reward-seeking motivation, i.e., drug dependence. This observation remains in concordance with findings of Steiner et al., who found that orexin deficiency reduces the risk of developing cocaine dependency [43]. Lopez et al. published results of a study assessing effects of OX1R antagonists in alcoholism; using the OX1R antagonist GSK1059865 in alcohol-addicted mice led to a reduction in their alcohol intake [44].

Orexin neurons project also to cholinergic neurons of the basal forebrain. This population of cells is crucial for performing multiple cognitive tasks. It seems that orexin plays a stimulatory role for cognitive performance enhancements. Piantadosi et al. showed in rats that blocking the action of orexin by intra-basal forebrain infusion with an OX1R antagonist causes a decline in learning and cognitive tasks solving, while an infusion of orexin-A enhances cognitive performance [45]. Probably orexinergic stimulation provokes the release of acetylcholine in the basal forebrain, which in turn improves intellectual abilities. The actions of orexins probably influence also memory functions. Dang et al. recently discovered that orexin knockout mice have impaired spatial memory [46]. Mavanji et al. proved in a series of experiments that orexin-deficient mice develop memory impairments, which may be successfully treated with orexin supplementation, e.g., intra-hippocampal infusion [47]. Data collected by Zhao et al. suggest that orexins exert their positive impact on memory through stimulation of neurogenesis in the hippocampus [48]. There are clinical data suggesting that the orexin system may be involved in attention maintenance. Weinhold et al. observed an improvement of the attention in narcolepsy patients treated with intranasal orexin-A [49]. On the other hand, one of the adverse events noted in clinical trials with dual orexin receptor antagonist like suvorexant was the occurrence of an attention deficit [26].

Once again, summing up the actions of orexin described above in one sentence may lead to the following phrase: “Stay awake, be alert, attentive, and motivated to eat as much as it is possible and burn it as quickly as you can”.

## Hidden Pacemaker—the Influence of Orexins on the Autonomic System and Blood Pressure

The consequences of orexin actions (wakefulness, alertness, motivation, and appetite) keep us appropriately reactive to external stimuli, so we can escape from a lion or follow the game in the savannah (suppose we were still hunter-gatherers like we used to be 100,000 years ago). This readiness for action implies also one more effect—an impact on the autonomic nervous system.

The relation between the orexin and the autonomic nervous system has its anatomical and functional background. Recently, Dergacheva et al. discovered direct connections between orexin neurons and dorsal motor nucleus of the vagus nerve. Additionally, the authors described two types of orexinergic stimulation of the vagal neurons: excitatory and inhibitory. This new finding may suggest that there are two subpopulations of orexinergic neurons with different roles in the autonomic control of the heart [50]. Orexin neurons project also to other areas of the brainstem, e.g., the rostral ventrolateral medulla (RVLM). It was shown by Korim et al. that this connection is responsible for the release of epinephrine during stressful events like hypoglycemia [51].

The influence of orexin on the autonomic nervous system has been well described for the cardiovascular system. Xiao et al. performed a series of experiments in rats with stress-induced hypertension. Microinjections of orexin-A into the RVLM led to a significant increase in blood pressure and heart rate in both hypertensive and control rats. This autonomic activation was then reversed with direct microinjections of the OX1R- and OX2R-selective antagonists SB-408124 and TCS OX2 29, respectively, into the RVLM. Xiao et al. also proved that the orexin-induced autonomic reaction, i.e., the increase in blood pressure and tachycardia, is blunted by injections of neuronal nitric oxide synthase (nNOS) and soluble guanylate cyclase (sGC) inhibitors into the RVLM. The findings described in this publication show that orexin exerts a direct influence on the cardiovascular reactivity of the autonomic nervous system through nitric oxide (NO) transmission and sGC-dependent signaling [52]. As the study cited above shows an interaction between the orexin and the autonomic nervous system at the level of the brainstem, Beig et al. demonstrated a more peripheral action of orexins. The authors injected a selective OX1R (ACT335827) or OX2R (EMPA) antagonist intragastrically or intraperitoneally into rats exposed to novelty stress and observed changes in tachycardic, pressor, and locomotor responses to stress. The effects of selective orexin receptor antagonists were compared to the effect of almorexant, a dual orexin receptor antagonist. The OX1R antagonist reduced the pressor and cardiac response to stress, while OX2R antagonist reduced just the pressor response. All three components of stress reaction were reduced in the case of a simultaneous injection with both selective antagonists or in the case of an administration of almorexant. The authors also found that OX1R and OX2R are present in preganglionic sympathetic neurons. This study shows that both orexins and both orexin receptors are involved in the control of autonomic reactions at the level of preganglionic sympathetic cells [53]. Martin et al. performed another interesting experiment analyzing the influence of orexin antagonists on the autonomic reaction in hypertensive rats exposed to air-jet stress. High doses of almorexant led to a reduction of the resting mean arterial pressure in hypertensive and control rats and attenuated the pressor and tachycardic response to stress. Selective blockade of OX1R resulted in a decrease of the resting arterial pressure and a smaller increase in heart rate after stress exposition, with no effect on the pressor reaction to stress. This study shows that orexin has a meaningful role in the response to stress in both hypertensive and control rats and that both orexin receptor types are involved in that reaction [54]. Yun and colleagues recently demonstrated that the involvement of the orexin system in stress response is even more profound. A typical hormonal reaction to stress—an increase in serum levels of adrenocorticotropic hormone (ACTH)—was absent in stress-exposed mice pretreated with selective OX2R antagonist [55].

Orexins exert their impact on the autonomic nervous system also in terms of breathing control. It was shown by Williams and colleagues that orexin neurons are excited by acidification and increases in CO_2_ concentrations [56]. Sugita et al. found that orexin stimulates the brainstem respiratory circuitry that probably contributes to the initiation of the expiratory phase [57]. Fonseca et al. presented evidence that blockade of OX1R in an animal model causes an attenuated reaction to hypercapnia and hypoxia leading to a decrease in tidal volume and breathing frequency [58]. These findings raised concerns whether clinical application of orexin receptor antagonists in the therapy of insomnia will cause breathing disorders in patients with compromised respiration. Clinical trials involving subjects with chronic obstructive pulmonary disease and obstructive sleep apnea showed that dual orexin receptor antagonists do not have a negative effect on respiration during sleep in those populations [59, 60].

The relations between the orexin system and the autonomic nervous system raised questions about whether orexin is involved in the control of blood pressure and if this transmitter may be involved in the genesis of hypertension. Many experiments using direct intracerebral injection of orexin showed a significant increase in blood pressure values. First tests with direct central injections of orexin were performed roughly 1 year after the discovery of orexin. Shirasaka et al. demonstrated in 1999 that injection of orexin causes a significant increase in sympathetic activity, heart rate, and blood pressure [61]. Huang showed that injections of orexin-A into the RVLM phenotypically resulted in an increase in blood pressure and heart rate that was attenuated by an OX2R antagonist, while an OX1R antagonist reduced only the increase in blood pressure. Orexin stimulation resulted also in the depolarization of adrenergic and noradrenergic RVLM neurons. This study shows that orexins exert their cardiovascular effect mainly through OX2R [62]. Intrathecal and intra-RVLM injections of orexin-A also lead to increased sympathetic activity and have a significant blood pressure effect that is reversed by orexin receptor antagonists [63, 64]. Just recently, Li et al. presented evidence for a significant pressor effect of orexin injection into the dorsomedial hypothalamus of rats, leading to a significant increase in arterial pressure values [65•].

Results of these orexin injection studies strongly suggest that actions of this neurotransmitter lead to increased sympathetic activity resulting in elevated blood pressure and heart rate. Despite these compelling studies, it should not be forgotten that injection of orexin does not mirror the physiological situation, e.g., due to a much higher local concentration of orexin compared with naturally occurring conditions. Another possibility to clarify the role of orexins in the control of blood pressure is to study the orexin system in hypertension models. Jackson et al. performed an experiment with the orexin receptor antagonist almorexant given to hypertensive mice (BPH/2J mice). In that study, the blockade of orexin receptors resulted in a significant reduction of the arterial pressure [66••]. More recently, Huber et al. analyzed in rats the activity of orexin neurons in salt-sensitive hypertension. Animals with salt-sensitive hypertension (Dahl S rats) were characterized by higher levels of OX1R and OX2R mRNA in the paraventricular nucleus suggestive of an increased activity of orexin neurons. Moreover, usage of an OX1R antagonist in those animals resulted in a significant drop in blood pressure [67]. Clifford et al. found that spontaneously hypertensive rats have more orexinergic neurons in the hypothalamus compared with normotensive animals [68]. Lee et al. obtained similar results, finding a higher number of orexinergic neurons in hypertensive rats correlated with increased orexinergic input to the RVLM and increased nitric oxide signaling [69].

There is a body of evidence suggesting that orexins are very important players in the control of blood pressure. It seems that they are “back-seat drivers,” hidden behind adrenergic and noradrenergic neurons of the brainstem and the peripheral autonomic nervous system, although exerting a powerful impact on the final blood pressure values. Nevertheless, all those data come from animal models and experimental studies. Clinical data on the relationship between the orexin system and blood pressure or hypertension are scarce.

Narcolepsy type I may serve as a clinical “model” of complete or almost complete lack of centrally acting orexins. If one simply transferred laboratory data into a clinical setting, one would expect that lack of orexinergic transmission (proven to increase arterial pressure values and presumably involved in the genesis of hypertension) would result in low blood pressure values or a reduced hypertension frequency in subjects with narcolepsy. Despite these expectations, Jennum et al. recently found that the prevalence of cardiovascular problems including hypertension in patients with narcolepsy is higher compared with that in controls. It is not clear whether this is a direct result of the pathological background of the disease (lack of orexin neurons, deficiency of orexin) or of other clinical features of narcolepsy (obesity, less intense physical activity, disturbed nocturnal sleep) or maybe this is a consequence of therapy with stimulants [70]. Clinical data are even more confusing. Donadio et al. discovered that, compared to healthy subjects, patients with orexin deficiency have lower levels of autonomic nervous system activity (as measured by muscle sympathetic nerve activity), lower blood pressure, and a decreased heart rate. The heart rate was correlated with the detected orexin level, while there was no such correlation for blood pressure. Additionally, subjects with lack of orexin had lower blood pressure than patients with detectable orexin levels [71]. These findings are in line with data regarding the role of orexin in generating sympathetic activity and increasing arterial pressure. Assessments of sleep-related changes in sympathetic activity and blood pressure values reveal even more confusing data. Nocturnal recordings show physiological changes of sympathetic activity through sleep stages although without the normal nocturnal decrease of blood pressure (“non-dipping” pattern) [72]. The last finding has been previously observed in two other studies. Grimaldi et al. described a normal 24-h rhythmicity of blood pressure and heart rate in patients with narcolepsy but with a notably blunted nocturnal decrease in arterial pressure (“non-dipping” pattern) [73]. The authors acknowledged that altered nocturnal changes of blood pressure were accompanied by other pathologic sleep phenomena like sleep fragmentation or presence of periodic limb movements in sleep. Dauvilliers et al. also described lack of nocturnal blood pressure dipping [74]. On the other hand, when a group of patients with narcolepsy was compared to subjects with insomnia, with no significant difference in sleep architecture between the groups, it appeared that there is no significant difference between the groups in terms of the prevalence of “non-dipping” [75]. That may suggest that the “non-dipping pattern” observed in narcolepsy may be a result of a disordered sleep (one of symptoms of the disease) and not of the orexin deficiency. The same research group showed that the presence of “non-dipping” pattern is not related to the orexin level in patients with narcolepsy [76].

So far, no unambiguous clinical evidence for the role of orexin in the control of blood pressure in humans was found on the basis of observations in narcolepsy patients. We do not have any data about the effect of pharmacological interventions targeting the orexin modulation of the arterial pressure. There are two studies with orexin-A being used intranasally by subjects with narcolepsy. Although authors of both papers described changes in sleep architecture, wakefulness, and cognitive performance, no information was published on any pressor effect of the drug [49, 77]. Suvorexant—the dual orexin receptor antagonist—went through a series of clinical trials and now is approved for the treatment of insomnia in the USA but no data regarding its action on the cardiovascular system has been published. Kuriyama and Tabata in their review on the clinical effects of suvorexant do not describe any significant impact on the cardiovascular system [78].

## Conclusions

Data collected so far suggest that orexins control functions of the cardiovascular system through stimulation of sympathetic neurons within the central nervous system. Both orexin receptors are involved in this control, and pharmacological interventions must be targeted at OX1R and OX2R to reach maximal effectiveness. Results of studies performed in animal models show that orexin intrinsically increases sympathetic tone and, thereby, elevates blood pressure levels. Nevertheless, the conclusion that orexin antagonism would solve problems regarding high arterial pressure is far too simplistic (although very tempting). First, data on the clinical effect of the anti-orexinergic action in humans in terms of cardiovascular control are still missing. Second, orexin transmission is involved in numerous phenomena that directly or indirectly influence cardiovascular function in humans, e.g., food intake, energy expenditure, motivation, and motor activity.

Studies on the clinical relevance of orexin led to the introduction of a new class of drugs for the therapy of insomnia: the orexin receptor antagonists (with one of them—suvorexant—actually approved for therapeutic use). It is still a promising field for the development of new drugs acting on the cardiovascular system, but at the moment, more translational studies are required.
